# Supplementary material for: SARS-CoV-2 Specific Nanobodies Neutralize Different Variants of Concern and Reduce Virus Load in the Brain of h-ACE2 Transgenic Mice
Source: Viruses. 2024 Jan 25;16(2):185. doi: 10.3390/v16020185 (PMC10892724; doi:10.3390/v16020185)
Supplement: Supplementary file 1 [file viruses-16-00185-s001.zip › viruses-2812528-supplementary.pdf]

Supplementary Materials

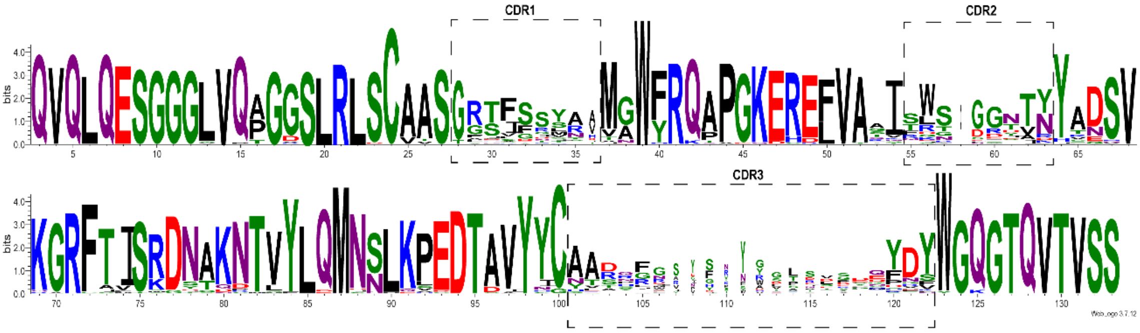

Figure S1. Sequence Logo Plot of a diverse repertoire of SARS-CoV-2 Nbs. Logo representation of amino acid multiple sequence alignments of the 43 unique Nbs selected after biopanning with RBD or S-2P proteins. The height of symbols indicates the relative frequency of each amino acid at that position.

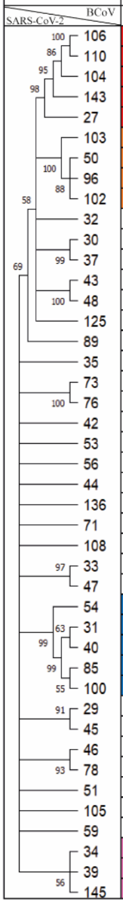

NJ Tree nucleotide sequences	Nb-ID	IMGT/V-QUEST			IMGT/DomainGapAlign		CDR3 Length	CDR3 Sequence	pI	Biopanning Antigen	Screening PE/PEE (S-2P)	Screening PE/PEE (RBD)	EC50 (nM) (Spike)	EC50 (nM) (RBD)
		V-GENE	D-GENE	J-GENE	V-GENE	J-GENE								
BCoV / SARS-CoV-2	10M	3S53*01	4*01	4*01	3S3*01	4*01	17	AAAYAPYTDYHPVEYVY	7.09	RBD	+/ND	+/ND	20	174.7
106	106	3S53*01	6*01	4*01	3S53*01	6*01	12	**YARNFVGRNPDV**	7.96	RBD	+/+	+/−	3.79	10.5
110	110	3S53*01	6*01	4*01	3-3*01	4*01	17	**AVARSWSDLDFENGYDY**	6.35	RBD	+/+	+/−	1.93	0.62
104	104	3S53*01	7*01	4*01	3S53*01	6*01	12	**YARNFVGHNPDV**	6.69	RBD	+/+	+/−	0.38	0.42
143	143	3S53*01	3*01	4*01	3S53*01	6*01	12	YARNFIGDNPDV	7.97	RBD	+/+	+/−	ND	ND
27	27	3S53*01	6*01	4*01	3S53*01	6*01	12	**YARNFVGDDFNL**	7.83	Spike	+/+	+/−	0.35	0.69
103	103	3S53*01	4*01	4*01	3S53*01	6*01	11	NADHFLDQFDS	6.12	RBD	+/+	+/−	ND	ND
50	50	3S53*01	4*01	4*01	3S53*01	6*01	11	NADHFLDQFDS	5.71	Spike	+/+	+/−	ND	ND
96	96	3S53*01	4*01	4*01	3S53*01	6*01	11	SADHFLDQFDS	6.70	Spike	+/+	+/−	ND	ND
102	102	3S53*01	4*01	4*01	3S53*01	6*01	11	SADHFLDQFDS	7.05	RBD	+/+	+/−	ND	ND
32	32	3S53*01	6*01	7*01	3S3*01	4*01	15	**NEARVYDSNWYPRDY**	8.98	Spike	+/+	-/-	0.012	neg
30	30	3S53*01	3*02	7*01	3S3*01	4*01	11	**NYHNSYYGSDY**	7.81	Spike	+/+	+/−	5.84	ND
37	37	3S53*01	2*01	7*01	3S3*01	4*01	11	**NYHNYYYGLDF**	6.64	Spike	+/+	+/−	3.36	0.04
43	43	3S53*01	2*01	4*01	3S53*01	4*01	13	**HFRQIRANAHDVY**	7.85	Spike	+/+	+/−	0.47	1.02
48	48	3S53*01	2*01	4*01	3S53*01	4*01	13	**HFRQIRAIADDVY**	8.98	Spike	+/+	+/−	0.56	1.78
125	125	3S53*01	2*01	4*01	3S53*01	6*01	10	**YARDLLQHPF**	6.00	RBD	+/+	+/−	12.04	ND
89	89	3S53*01	6*01	4*01	3S53*01	2*01	11	**AAKTRGVYGTN**	7.54	Spike	+/+	+/−	14.65	ND
35	35	3-3*01	4*01	4*01	3S1*01	4*01	15	**ASDLRMYEGDWYGGY**	6.27	Spike	+/+	-/-	23.77	neg
73	73	3-3*01	2*01	4*01	3-3*01	2*01	18	ARHSRPYDSGRKVGDYAY	7.81	Spike	+/+	-/-	ND	ND
76	76	3-3*01	2*01	4*01	3-3*01	4*01	18	ATDPGNFGLPYSELEYNY	6.81	Spike	+/+	+/−	ND	ND
42	42	3-3*01	6*01	4*01	3S2*01	4*01	15	AARNGYSPFVRSYDY	6.81	Spike	+/+	-/-	ND	ND
53	53	3-3*01	8*01	4*01	3-3*01	4*01	15	**AAGGRISNEGFKPDY**	8.52	Spike	+/+	-/-	103.6	neg
56	56	3-3*01	2*01	4*01	3-3*01	4*01	22	AARYAAIGSPYWKLTTPSEYEY	8.50	Spike	+/+	-/-	ND	ND
44	44	3-3*01	4*01	4*01	3-3*01	4*01	19	**AADPFFRVIAYSDPKRYSY**	6.64	Spike	+/+	+/−	50.44	16.74
136	136	3-3*01	2*01	4*01	3-3*01	4*01	18	**AAYEGIRYSSTRADFYNY**	7.80	RBD	+/+	+/−	174.7	13.45
71	71	3-3*01	1*01	4*01	3-3*01	4*01	17	**AVAREWSDLDFRDGYDY**	7.80	Spike	+/+	+/−	0.14	1.63
108	108	3-3*01	6*01	4*01	3-3*01	4*01	11	AATGDGGSYDY	7.01	RBD	+/+	+/−	ND	ND
33	33	3-3*01	5*01	4*01	3S3*01	4*01	18	**AVTRRRFVTLRVPDDYDK**	8.99	Spike	+/+	-/-	0.025	neg
47	47	3-3*01	2*01	4*01	3-3*01	2*01	18	AATRRRYHTLRVSGDYDN	6.71	Spike	+/+	-/-	ND	ND
54	54	3-3*01	2*01	4*01	3-3*01	4*01	17	AASQFGSGADYSEQYDY	7.94	Spike	+/+	-/-	ND	ND
31	31	3-3*01	2*01	4*01	3S5*01	4*01	17	**ATSQFGSGTSDSEQYDY**	8.55	Spike	+/+	+/-	ND	137.4
40	40	3-3*01	4*01	4*01	3S5*01	4*01	17	**ATSQFGSGTSDSEQYDY**	8.55	Spike	+/+	-/-	1.06	169
85	85	3-3*01	4*01	4*01	3-3*01	4*01	17	**ATSQFGSGTSDSEQYDY**	8.55	Spike	+/+	-/-	0.81	neg
100	100	3-3*01	4*01	4*01	3-3*01	4*01	17	**ATSQFGSGTSDSKQYDY**	8.93	RBD	+/+	+/−	1.92	neg
29	29	3-3*01	2*01	4*01	3S1*01	4*01	18	**AASSWDSRGSSNYVHYDY**	8.49	Spike	+/+	+/-	0.05	55.89
45	45	3-3*01	2*01	4*01	3-3*01	4*01	18	**AASSRDSRGTSTYASYDY**	8.52	Spike	+/+	-/-	1.84	neg
46	46	3-3*01	5*01	4*01	3-3*01	4*01	20	**AATALGYRYASLESRNEYRY**	7.79	Spike	+/+	-/-	0.38	neg
78	78	3-3*01	5*01	4*01	3-3*01	4*01	20	**AATGLGYRYAVLESRSEYRY**	8.52	Spike	+/+	-/+	0.53	16.59
51	51	3-3*01	1*01	4*01	3-3*01	4*01	19	**AARYPGKWGELPTDTRFDF**	8.85	Spike	+/+	-/-	4.42	neg
105	105	3-3*01	2*01	4*01	3-3*01	4*01	18	**AADRGESYYYSRPSEYTY**	6.02	RBD	+/+	+/−	1.08	4.69
59	59	3-3*01	4*01	4*01	3-3*01	4*01	20	AADKGMGYYRDAALASWYDY	8.55	Spike	+/+	-/-	ND	ND
34	34	3-3*01	1*01	4*01	3S3*01	4*01	19	ATDPGNFGLPNSELEFNY	6.45	Spike	+/+	+/−	ND	ND
39	39	3-3*01	5*01	4*01	3S3*01	4*01	19	**CAADPGNFGVPIEYFMFDY**	6.27	Spike	+/+	+/−	0.14	0.29
145	145	3-3*01	5*01	4*01	3-3*01	4*01	19	**CAADPGNFGVPIEHFMYDY**	7.96	RBD	+/+	+/−	0.81	0.69

Figure S2. Characterization of isolated Nbs against SARS-CoV-2. Phylogenetic analysis of Nbs selected after the biopanning of two Nb-libraries. Nanobodies can be clustered in four groups (a, b, c and d, highlighted by the red, orange, blue and pink branches, column 1). Nanobodies in all

clusters were selected with both antigens, indicating a good selection strategy. Cluster A was supported with a 98% bootstrap value and constituted a monophyletic group of binders directed to RBD (Nb-27, Nb-143, Nb-106, Nb-104 and Nb-110) with 69% to 91% AA homology, all with a CDR3 of 12 AA long except for Nb-110 that has a longer CDR3 of 17 AA. Cluster B was supported with a 100% bootstrap value and included four binders directed to RBD, which possess almost the same CDR3, suggesting they belong to the clonal expansion of the same B cell. Cluster C was composed of five Nbs with similar lengths in the CDR3 and an average homology of 91%. Cluster D had a low bootstrap value of 56%, however the homology was high (82.4 to 91.2%) and all Nbs had a 19 AA long CDR3. Germline origin according to IMGT/V-QUEST (columns 3 to 5) and the IMGT/DomainGapAlign programs (columns 6 and 7) show gene segment (V), diversity gene segment (D), and junction gene (J) for each Nb. Same colors are used for similar VDJ origin. CDR3 composition and length are depicted in column 8 and 9. CDR3s marked in bold correspond to the Nbs selected for further characterization. Isoelectric point values are shown in column 10. The antigen used for panning and binding to the SARS-CoV-2 proteins is depicted in columns 11 to 13. The binding activity of each Nb to the S-2P and RBD proteins (half-maximal effective concentration, EC_{50} in nM), is shown in columns 14 and 15. ND: not determined.

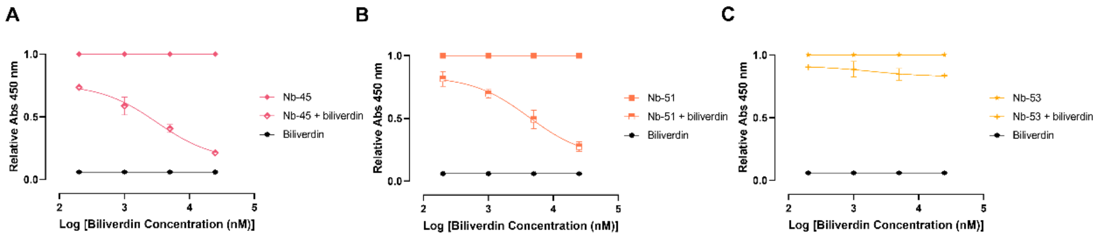

Figure S3. Biliverdin decreases the binding of non-RBD binders to S-2P protein. Relative dose-response curves for Nb-45 (a), Nb-51 (b) and Nb-53 (c) in the absence or presence of different concentrations of biliverdin.

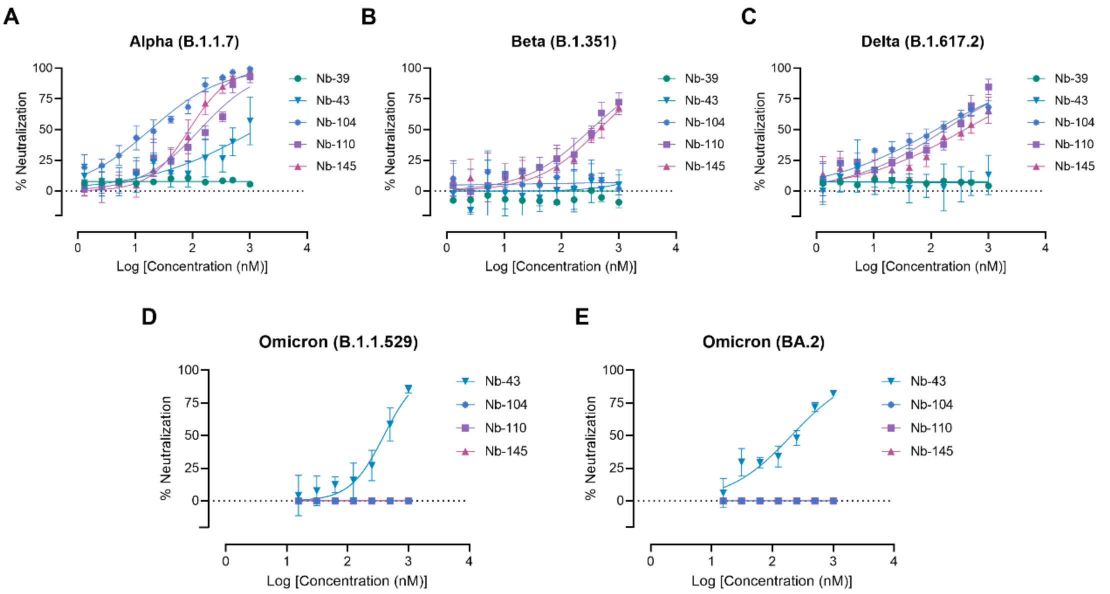

Figure S4. Neutralization titers of SARS-CoV-2 strains *in vitro* by RBD binders. Neutralization curves and IC_{50} of the RBD binders for (a) Alpha (B.1.1.7); (b) Beta (B.1.351); (c) Delta (B.1.617.2) and (d and e) Omicron (B.1.1.529 and BA.2) variants of SARS-CoV-2 measured by pVNT. None of the RBD binders were able to neutralize the XBB and XBB.1.5 variants. Data are shown as the mean (n = 3) with SD. The IC_{50} was calculated by fitting the inhibition from serially diluted Nbs to a sigmoidal dose-response curve. The experiment was performed in triplicate.

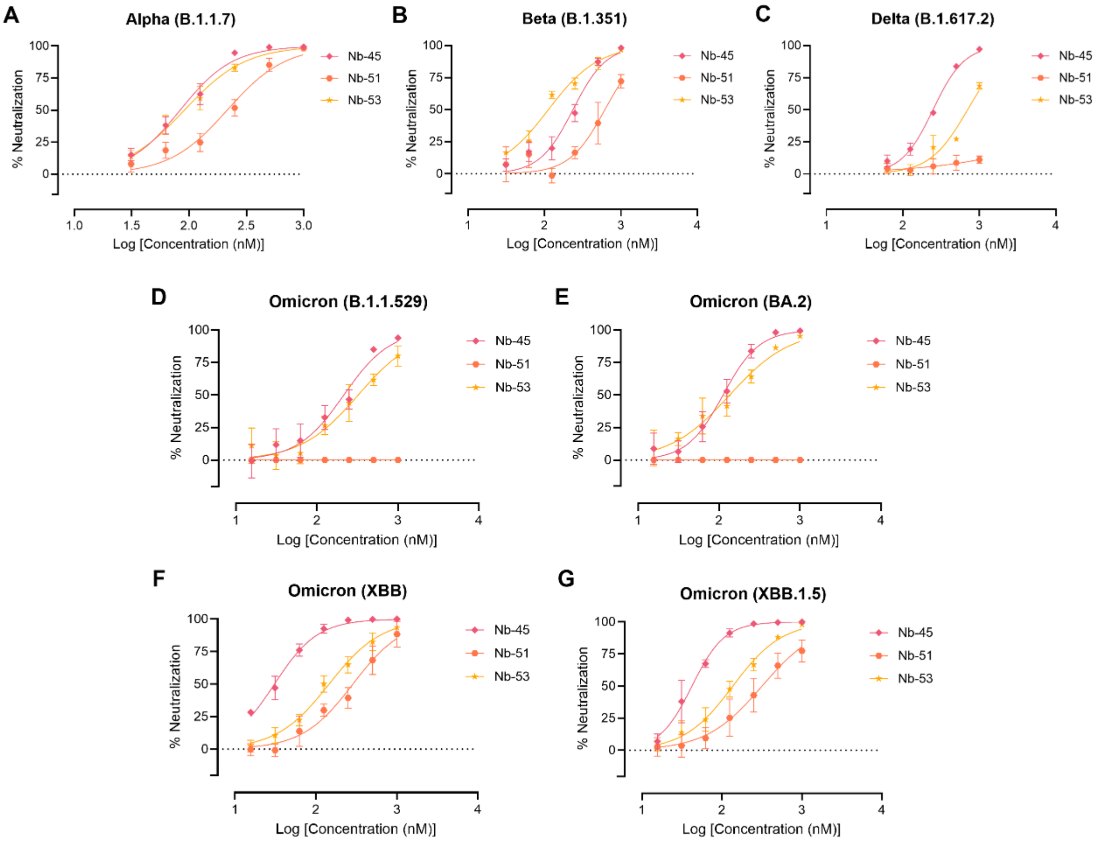

Figure S5. Neutralization titers of SARS-CoV-2 strains *in vitro* by non-RBD binders. Neutralization curves and IC_{50} of the non-RBD binders for (a) Alpha (B.1.1.7); (b) Beta (B.1.351); (c) Delta (B.1.617.2) and (d-g) Omicron (B.1.1.529, BA.2, XBB and XBB.1.5) strains of SARS-CoV-2 measured by pVNT. Data are shown as the mean (n = 3) with SD. The IC_{50} was calculated by fitting the inhibition from serially diluted Nbs to a sigmoidal dose-response curve. The experiment was performed in triplicate.

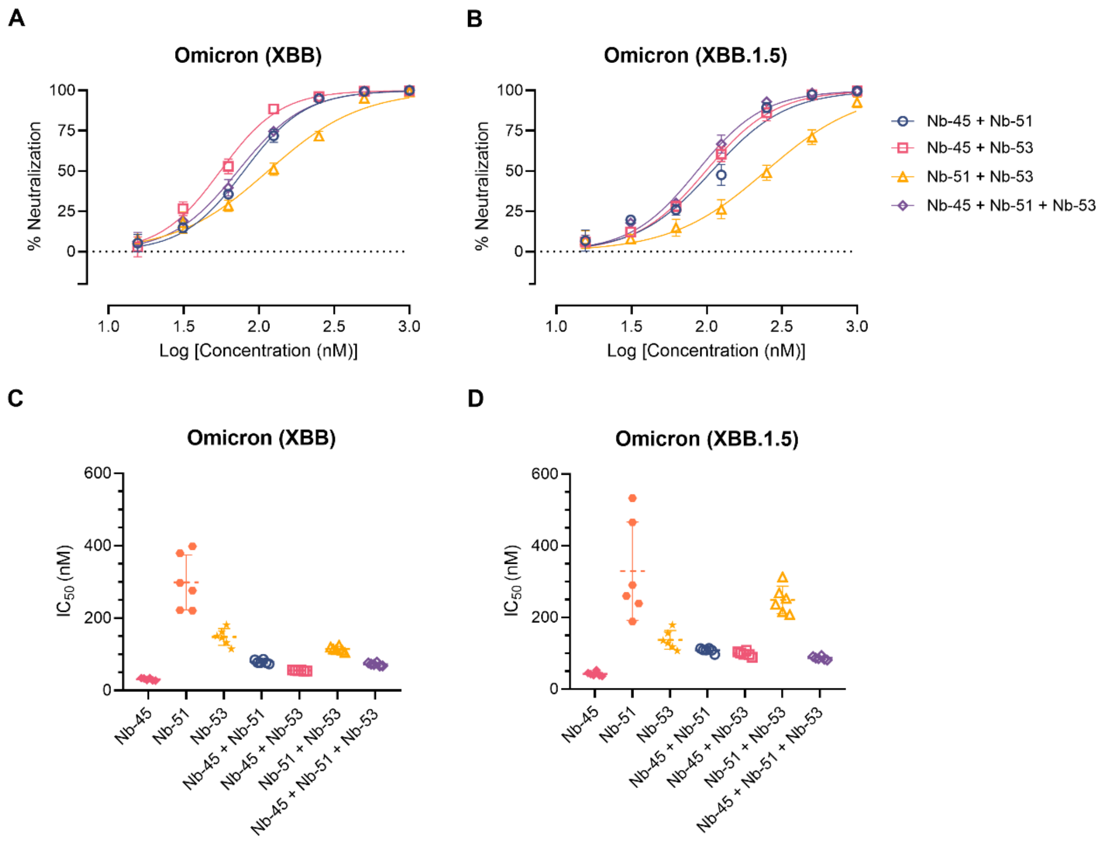

Figure S6. Enhancement of neutralization potencies against Omicron XBB subvariants using Nanobody cocktails. (a) Percentage of neutralization exerted by a mixture of two or three Nbs against the XBB Omicron variant; (b) Similar experiments than in A performed with the XBB.1.5 Omicron variant; (c and d) Comparison of IC_{50} values of Nbs combinations vs. individual Nbs for the B.1.1.529 Omicron variant and the BA.2 Omicron variant.
